# Medicine

**Published:** 1902-06

**Authors:** G. Parker


### Progress of the Medical Sciences.

### MEDICINE.

**Diseases of the Stomach.**—While it has become possible from improved methods to diagnose many gastric affections with fair certainty, others remain which are recognised with difficulty. **Dyspepsia due to adhesions** is one of these. It is so easy to mistake it for simple dyspepsia from bad food or from hyperchlorydria, for latent ulcer of the stomach, for hysteria and neurasthenia, and for neuralgia. There have been however a number of papers recently on the subject by W. Calwell, Kauffman, Bird, and Hale White, from which important indications may be learnt. The condition may be due to previous ulcers, affections of the bile ducts, syphilis, or injuries. It is essentially chronic. Sometimes the stomach will accommodate itself to the local obstruction and, if care is taken to avoid taxing it with bulky food, it will manage to pass on its load without much pain. Usually indeed, as Bird[1] and W. Calwell[2] point out, there are attacks of pain definitely felt as coming from one spot, varying from a dull ache to an unbearable agony. This pain does not follow at once after a meal, but from thirty minutes to several hours later. The patient is hungry, but afraid to eat, and above all the pains are started or aggravated by movement. On lifting a weight or stretching they become stabbing or shooting in character; but when lying in bed and on a light diet they may almost entirely disappear, and are often lessened by wearing a bandage. Vomiting may follow the onset of the pain, and from this and the fear of eating, emaciation may be considerable. If there is no blood, excess of acid, or mucus in the vomit, and the symptoms have continued for a long while, the probability of adhesions is great, especially if there is a history of gastric ulcer formerly. Mayo Robson and Moynihan[3] say if the stomach be distended with gas the cavity will extend further to the right and higher than normal if adhesions are present. Hale White[4] remarks that above 45 per cent. of ulcers form adhesions to neighbouring organs, and besides the troubles we have considered, they may produce dilatation, with periodical vomiting of long-retained food, coated tongue, and other well-known symptoms. Even without this the adhesions may cause local tenderness and an indefinite fulness where the organs are matted together, compelling us to consider the possibility of a malignant growth. Of course it is also necessary to exclude tabetic crises, neuralgia, gallstone

[1] *Am. J. M. Sc.*, 1901, cxxii. 104. [2] *Dublin J. M. Sc.*, 1901, cxii.
[3] *Diseases of the Stomach and their Surgical Treatment*, 1901, p. 226.
[4] *Lancet*, 1901, ii. 1471.

colic, referred pains from floating kidneys, and other causes. Another occasional result of adhesions is perforation. Thus, in a well-marked case of seven years' duration the patient washed her hair, and the strain from the arms in rubbing her head appears to have torn through the adhesions, causing instantaneous pain with collapse, peritonitis and death in forty hours. The pains of ordinary adhesions may reduce the patient to a wretched helpless invalid, and the question of operation may have to be faced. Sometimes the results are brilliant; but adhesions of the posterior wall are most difficult to reach, and Hale White adds two reasons why a guarded prognosis should be given before operating. One is the possibility of adhesions reforming, and the other is that patients who have suffered pain so long undergo a psychical change which may lead afterwards to the development of pains in the old spot which may be hysterical or neuralgic in nature.

A special form of adhesion is sometimes seen with a small hernia in the linea alba. The dragging pains commencing after a fall or strain, increased by exertion and disappearing with rest in bed in otherwise healthy people, are most characteristic, and the small gland-like protrusion in the middle line, no larger perhaps than a hemp seed, is not easily forgotten when once felt. Like other herniæ, it may be reducible or incarcerated; but small as it is, the effect on the stomach is as serious as many of the adhesions due to old ulcers.

**Syphilitic affections of Stomach.**—Another group of stomach affections rarely recognised is that due to syphilis. Soltau Fenwick[1] divides them into gummata, endarteritis, and chronic inflammation of the mucous membrane. Besides the irregular ulcers due to gummata, others are the result of syphilitic endarteritis, which are difficult to distinguish clinically from so-called simple ulcers, though much less common. He estimates indeed that not more than 5 per cent. of all cases of gastric ulcer are connected with syphilis. However it is important to recognise them, for they are most resistant to the ordinary remedies for ulcer. Among other peculiarities it should be noted that they generally occur in males from twenty-five to forty years of age who are markedly anæmic and emaciated. The pain is especially severe, and is worse at night. Vomiting too is excessive, but hemorrhage is rare. They yield quickly to mercury and iodides, but are apt to relapse. Simon Flexner[2] collected some fifteen cases of syphilis of the stomach, five of which were of the inherited form and nine of the acquired. From cancer the diagnosis is difficult; but hæmatemesis and the presence of a tumour would be most likely due to cancer. Specific treatment too in a few days actually increases the pain of cancer, while its beneficial

[1] *Lancet*, 1901, ii. 835. [2] *Am. J. M. Sc.*, 1898, cxvi. 424.

results are quickly shown in syphilitic ulcer. The gastric crises of tabes may need to be distinguished; but they occur at irregular intervals and do not follow the ingestion of food, and the patients have usually other symptoms of the disease. Such crises have also been noticed in rheumatoid arthritis.

**Pneumococcal** and **streptococcal gastritis** are not unknown. The former may be accompanied with a fibrinous exudation over the stomach walls, petechiæ on the skin, and a septicæmic state, and true duodenal and gastric ulcers occasionally occur in pneumonia, which are probably of toxic origin since pneumococci have not been found in them. Three cases of the latter have been recently reported by W. Cayley,[1] O. A. Schultze,[2] and L. A. Conner,[3] under the name of phlegmonous gastritis. There may be rigors, vomiting, violent pain, sometimes dilatation of the stomach, and vomiting of pus. The streptococci are found in the submucosa and muscular coats, and quantities of infiltrating pus are formed.

**Ulcers** are occasionally produced by the **diphtheria bacillus,** as in a case recorded by W. R. Stokes,[4] where the lesion was situated on the lowest part of the greater curvature towards the pylorus, and the mucous coat and part of the submucosa was destroyed. Usually the stomach is able to destroy the bacilli; but when its secretory power is weakened true diphtheria of the stomach may occur, of which instances have been noticed by Schoedel and others.

**Ulcer of the œsophagus** is another little-recognised affection which is important, as the hemorrhage may lead to an erroneous diagnosis of pulmonary disease or of true gastric ulcer. Varicose ulcers indeed in cirrhosis of the liver, with their profuse sudden venous hemorrhage, are well known, and need not detain us except to remark on the danger of giving drugs which raise the blood pressure in such cases. M. I. Knapp[5] believes that simple ulcers are very common, and that the pain of œsophagitis felt between the shoulder blades and behind the sternum is too often dismissed by the mystic and euphonious phrase, "a reflex pain." An important symptom of ulcer is spasm of the œsophagus when a soft tube is passed. This spasmodic contraction may be sufficient to stop the passage of a tube entirely, and may be caused either by an active ulcer or by the cicatrix of a newly-healed one. Too often such a condition is put down to hysteria, and indeed the difficulty may be due to want of dexterity in the operator. The hemorrhages may occur at intervals for years, and the blood is bright red in colour if it is ejected at once. The writer refers to at least one patient who

[1] *Lancet*, 1902, i. 227. [2] *Med. Rec.*, 1901, lx. 877. [3] *Ibid.*
[4] *Johns Hopkins Hosp. Bull.*, 1901, xii. 209.
[5] *Med. Rec.*, 1902, lxi. 334.

had been under treatment for supposed phthisis, but had merely ulcer of the œsophagus, with an excess of organic acids and various micro-organisms flourishing in the stomach contents. In this case too the temporary spasmodic stricture on passing the stomach tube was well marked. The ulcers seem often to occur in alcoholic patients; and even if the patient has phthisis, ulcer of the œsophagus may be occasionally the cause of a hemorrhage. On the other hand it is of course most important not to mistake a pulmonary hemorrhage for one from the œsophagus.

**Acute dilatation of the stomach** was the subject of an interesting debate at the Royal Medical and Chirurgical Society, and of several later papers. Campbell Thompson[1] collected forty-nine cases, some of which occurred after surgical operations, injuries, or indiscretions of diet, while others accompanied various diseases; but in seven no predisposing cause could be found. There is usually a sudden onset, violent and copious vomiting, perhaps with remissions, collapse, abdominal pain and tenderness, distension of the gastric region with splashing sounds and fluctuation, but rarely if ever visible peristalsis. The dilatation and the great secretion of fluid in the stomach do not always occur together, and the former is, he thinks, due primarily to a nerve paralysis, though the increased secretion may increase dilatation when it has once commenced. There is clearly no pyloric obstruction, for nothing of the kind is found *post-mortem*, and a variable portion of the duodenum is also dilated. Nor is the existence of vomiting against paralysis and in favour of obstruction, for physiologists have shown that vomiting can take place when the stomach is replaced by a bladder, provided the cardiac orifice is open and the abdominal muscles and diaphragm are intact. Albrecht[2] and Ewart[3] think there is obstruction at the junction of the duodenum and jejunum from constriction by the superior mesenteric arteries, following displacement of the intestines towards the pelvis; but it appears that considerable force is required to cause constriction, and moreover the intestines are not found to be displaced in all cases, and the point where the dilatation ends is variable. Thus it is much more probable that paralysis is the primary cause of the dilatation, and Box and Wallace[4] show that the stomach contents are then prevented from flowing downwards by the pressure of the organ on the duodenum where it crosses the vertebral column. This can be demonstrated on the cadaver even after the bowel has been cut through anywhere to the left of the spine. Hence they recommend that the patient should be placed in the prone position, or at least on the right side. In fact, as Ewart says, if there is any fear of an attack, we

1 *Acute Dilatation of Stomach*, 1902.
2 *Arch. f. path. Anat.* [etc.], 1899, clvi. 285.
3 *Lancet*, 1901, ii. 1228, 1449. 4 *Ibid.*, 1259.

have another reason for not allowing the patient to remain always on his back. When acute dilatation has taken place food should be given by the rectum, the fluid in the stomach should be drawn off by a tube, and hypodermics of strychnine freely administered. If the quantity of fluid lost from the organism by the secretion into the stomach is very great, saline injections per rectum are desirable. Ewart suggests the knee and elbow position if the patient is not too ill; but the affection is rapidly fatal in most cases, though it is probable that mild cases are often unnoticed.

Tetany in chronic dilatation has been variously ascribed to dehydration of the tissues, to an excess of H.Cl., or to a peptotoxin. Halliburton and McKendrick[1] have succeeded in partially isolating the poison. It causes a great fall of blood pressure and slows the heart when injected into animals. It has a marked acid reaction, and is soluble in alcohol or in saline solutions. Cassaed[2] obtained two extractives, one of which produced coma and the other caused convulsions. He inclines to the view that we have to do with a substance which has been partially peptonised and then absorbed.

G. PARKER.

### SURGERY.

[1] *Lancet*, 1901, i. 174. [2] *Brit. M. J.*, Epit., 1901, i. 73.

